# Performance Analysis of Physical Layer Security of Opportunistic Scheduling in Multiuser Multirelay Cooperative Networks

**DOI:** 10.3390/s17020377

**Published:** 2017-02-15

**Authors:** Kyusung Shim, Nhu Tri Do, Beongku An

**Affiliations:** 1 Graduate School of Smart City Science Management, Hongik University, Seoul 30016, Korea; kyusung@hongik.ac.kr; 2 Department of Electronics and Computer Engineering, Hongik University, Seoul 30016, Korea; dotrinhu@gmail.com; 3 Department of Computer and Information Communications Engineering, Hongik University, Seoul 30016, Korea

**Keywords:** physical layer security, opportunistic scheduling, cooperative relaying transmissions, maximal ratio combining, selection combining, secrecy outage probability

## Abstract

In this paper, we study the physical layer security (PLS) of opportunistic scheduling for uplink scenarios of multiuser multirelay cooperative networks. To this end, we propose a low-complexity, yet comparable secrecy performance source relay selection scheme, called the proposed source relay selection (PSRS) scheme. Specifically, the PSRS scheme first selects the least vulnerable source and then selects the relay that maximizes the system secrecy capacity for the given selected source. Additionally, the maximal ratio combining (MRC) technique and the selection combining (SC) technique are considered at the eavesdropper, respectively. Investigating the system performance in terms of secrecy outage probability (SOP), closed-form expressions of the SOP are derived. The developed analysis is corroborated through Monte Carlo simulation. Numerical results show that the PSRS scheme significantly improves the secure ability of the system compared to that of the random source relay selection scheme, but does not outperform the optimal joint source relay selection (OJSRS) scheme. However, the PSRS scheme drastically reduces the required amount of channel state information (CSI) estimations compared to that required by the OJSRS scheme, specially in dense cooperative networks.

## 1. Introduction

Physical layer security (PLS) techniques have been emerging as a robust solution to prevent information eavesdropping for future wireless networks, especially for cooperative relaying networks [1,2,3,4]. The underlying idea of PLS is to exploit the physical characteristics of wireless channels to securely transmit information between legitimate users [5]. More specifically, from the information-theoretic perspective, information can be confidentially transmitted if the main channels (the channels between legitimate users) and the eavesdropper channels (the channel from a legitimate user to an eavesdropper) can be managed or controlled so that the legitimate destinations can decode their information successfully while the eavesdroppers are not able to decode their overheard information. It is shown that PLS is on the cutting-edge of the technologies of security, particularly in wireless communications to prevent eavesdropping attacks [3,6]. In PLS, the maximum rate at which the information can be confidentially transmitted between legitimate users is termed the secrecy capacity. The secrecy capacity can be determined by the difference between the capacity of the main channel and that of the eavesdropper channel [5]. Additionally, the performance of PLS is also evaluated in terms of the secrecy outage probability (SOP). The SOP can be defined as the probability that the secrecy capacity falls below a predefined target secrecy rate [5].

Cooperative relaying transmissions have been recognized as an efficient method to improve the coverage and capacity of wireless networks and have been adopted in industry standards, e.g., the IEEE 802.16j standard for relay-based wireless access networks [7]. In the literature, uplink scenarios of multiuser multirelay cooperative networks can be described as multiple sources transmitting data to a single destination via the help of multiple relays [8]; and downlink scenarios can be described as a single source communicating with multiple destinations via multiple relays [9]. In such systems, opportunistic scheduling, i.e., a source (destination) communicates with only one scheduled destination (source) with the assistance of only one selected relay, has been recognized as an attractive scheduling method to improve system performance [8,9,10] since the time-varying nature of wireless channels is exploited.

While the cooperative relaying transmissions have the ability to increase the reliability, as well as the transmission range of wireless communications, it is also more vulnerable to attackers because the same information is transmitted twice (by a source and then a relay). PLS has been shown as a potential means to combat this issue.

Considering the downlink/uplink scenarios of wireless networks where a central entity (e.g., base station or access point) communicates with multiple users, existing works in the literature have shown that the performance of such networks will be better if the central entity chooses to communicate with the user having the best channel condition [11,12,13], particularly under the consideration of physical layer security (PLS) [14]. Furthermore, the performance improvements of the best user selection scheme (for downlink or uplink scenarios) have been demonstrated in cooperative relaying networks with or without considering PLS [15,16]. Owing to these facts, in this paper, we adopt the best source selection scheme together with the best relay selection scheme to improve the performance of multiuser multirelay cooperative networks under the consideration of PLS.

Next, we are going to elaborate on the applications of PLS into multiuser and/or multirelay cooperative networks.

In [16], considering both amplify-and-forward (AF) and decode-and-forward (DF) protocols, the authors proposed optimal relay selection protocols to help the improvement of the wireless security of a given source destination transmission against an eavesdropper. Furthermore, considering the single source destination pair, the authors in [17] extended to the case of multiple eavesdroppers; three opportunistic relay selection protocols with different required overhead information were proposed. Considering a downlink transmission from a base station to multiple destinations via the assistance of a DF relay in the presence of multiple eavesdroppers, the authors in [18] investigated the secrecy performance of the maximal ratio transmission scheme when the eavesdropper’s channel state information (CSI) is available and is not available at the relay, respectively. In [19], considering the DF protocol, the author assumed a destination using cooperative jamming (CJ) to improve the system performance. Then, the best relay is selected based on the achievable system secrecy capacity. Additionally, the authors optimized the transmit power of source, relay and destination, respectively. The authors in [20] studied a large-scale multi-input multi-output (LS-MIMO) relaying system. Specifically, en evaluation method of secrecy outage capacity was proposed for the case for which the CSI of the eavesdropper channel is not available and the CSI of the main channel is imperfect. In [21], assuming an uplink scenario where one selected legitimate user wants to transmit data to a base station while eavesdroppers attempt to intercept the legitimate transmission, two opportunistic schedulings with and without instantaneous CSI of eavesdroppers, respectively, were investigated. Next, considering a similar uplink scenario, but the base station is now equipped with multiple antennas, the authors in [22] proposed two low-complexity user selection schemes under different requirements of eavesdropper’s CSI. In [23], the impact of different diversity combining techniques, namely maximal ratio combining (MRC) and selection combining (SC) on the security of an uplink cooperative transmission, where a selected source transmits data to a destination via a multi-antenna relay, were studied. Recently, PLS in multiuser multirelay cooperative networks was investigated in [24,25]. In particular, the authors in [24] proposed that multiuser and multirelay selection schemes based on the set of relays can perfectly decode from the base station. The authors in [25] considered two criteria for source relay selection proposed by exploiting the direct links between sources and destination. Very recently, secure multiuser communications in wireless sensor networks were investigated in [26], where the switch-and-stay combining technique was adopted to reduce the scheduling complexity and extend the battery lifetime of the sensor nodes, while the transmit antenna selection technique and cooperative jamming were used to achieve satisfactory secrecy performance.

In this paper, we consider an uplink scenario of multiuser multirelay cooperative networks, where a selected source communicates with a destination via the help of a selected relay in the presence of one eavesdropper. Compared with the above related works, the key contributions of this paper can be summarized as follows.

- We propose a low-complexity, yet performance comparable, source relay selection scheme, named the PSRS scheme, by taking into account both the CSIs of the main and eavesdropper channels. Specifically, the PSRS scheme first selects the least vulnerable source, in other words the source that minimizes the channel gains of the source eavesdropper channels; then, the relay that maximizes the end-to-end system secrecy capacity for the given selected source is chosen. In addition, the eavesdropper is assumed to perform either the maximal ratio combing (MRC) technique or the selection combing (SC) technique to combine its overheard signals.
- In order to analyze the secrecy performance of the considered system, we derive new closed-form expressions of the system secrecy outage probability (SOP), which have not been reported in the literature. The developed analysis is verified by Monte Carlo simulation to confirm our correctness.
- From the numerical results, we show that the PSRS scheme achieves better secrecy performance than that of the random source relay selection scheme, but does not outperform the optimal joint source relay selection (OJSRS) scheme. However, the PSRS scheme significantly reduces the required amount of CSI estimations compared to that required by the OJSRS scheme, especially when the numbers of sources and relays are large.

The rest of the paper is arranged as follows. Section 2 introduces the system model and describes in detail the selection criterion of the PSRS scheme. Section 3 presents the developed analysis in terms of the SOP for the considered scenarios. Section 4 presents some illustrative numerical results, based on which insightful discussions are provided. Monte Carlo simulations are shown to corroborate the proposed analysis. Finally, Section 5 concludes the paper.

## 2. System Model

**Figure 1:**
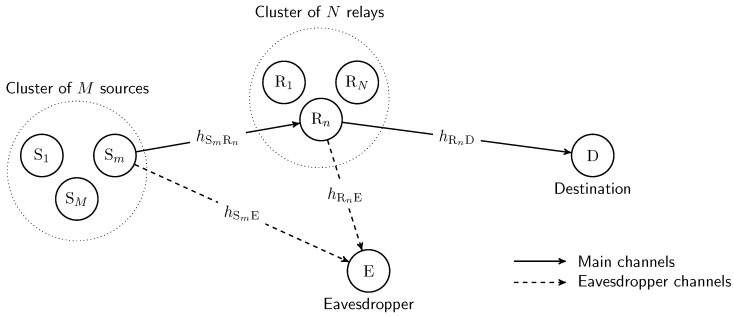
Schematic illustration of the multiuser multirelay cooperative networks with the presence of an eavesdropper.

Let us consider a multiuser and multirelay cooperative network, where a source communicates with a destination via the help of a relay with the presence of an eavesdropper as depicted in Figure 1. Specifically, the system is composed of a set of *M* sources $S=\{S_{m}|m=1,\ldots ,M\}$, a set of *N* relays $R=\{R_{n}|n=1,\ldots ,N\}$, one destination D and one eavesdropper E. We assume that the direct links between the users and the destination are not available due to the destination being out of the coverage area, or severe fading, or deep shadowing, as in [16,17,23]. Moreover, the data transmission from the source to the destination is carried out through the support of the decode-and-forward (DF) relays. Specifically, a time-division multiple-access scheme is used for orthogonal access, and we assume perfect synchronization in the network. The slot of one data transmission is divided into two sub-slots, which equal the time duration. In the first sub-slot, a selected source broadcasts its signal. In the second sub-slot, a selected relay perfectly decodes the received signal from the source and then forwards the signal to the destination. Considering the physical layer security of such a cooperative network, we assume that an eavesdropper monitors the communication between legitimate users, i.e., the user and destination. We further assume that all nodes are equipped with an omnidirectional antenna and operate in half-duplex mode. All wireless links are assumed to undergo independent and identical distributed (i.i.d.) Rayleigh block flat fading. In order to provide a comprehensive analysis of the PLS of the considered relaying system, we assume that the required channel state information (CSI) is available, as in [16,17,25].

In the following part of the paper, let $h_{\mathrm{XY}}$ denote the fading coefficient of the X→Y channel, where X∈S∪R and Y∈R∪{D,E}. Under the assumption of i.i.d. Rayleigh fading, $h_{\mathrm{XY}}$ can be modeled as i.i.d. complex Gaussian random variables with zero-mean and variance $\lambda_{\mathrm{XY}}$. Additionally, let $n_{Y}$ denote the additive white Gaussian noise (AWGN) at node Y with zero-mean and variance $\sigma_{Y}^{2}$.

### 2.1. Communication Process

The communication process is carried out in two phases, namely the broadcasting phase and the relaying phase, which are conducted in the first and second sub-slots, respectively. Without loss of generality, in a certain transmission slot, we suppose that the source $S_{m}$ has been selected to transmit its data, and the relay $R_{n}$ has been chosen to help the selected source.

#### 2.1.1. Broadcasting Phase

In the broadcasting phase, $S_{m}$ transmits a normalized signal *s*, i.e., $E[|s{|}^{2}]=1$, where E[·] denotes the statistical expectation operator, with transmit power $P_{S_{m}}$. Thus, the received signal at $R_{n}$ can be expressed as:

(1) $$y_{S_{m}R_{n}}=\sqrt{P_{S_{m}}}h_{S_{m}R_{n}}s+n_{R_{n}},$$

where $h_{S_{m}R_{n}}∼\mathrm{CN}\left(0,\lambda_{S_{m}R_{n}}\right)$ and $n_{R_{n}}∼\mathrm{CN}\left(0,\sigma_{R_{n}}^{2}\right)$; herein, $\mathrm{CN}(0,\sigma^{2})$ denotes a circular symmetric complex Gaussian variable with zero-mean and variance $\sigma^{2}$.

Meanwhile, the eavesdropper can intercept the source signal due to the broadcast nature of wireless communications. Thus, the overheard signal at E can be written as:

(2) $$y_{S_{m}E}=\sqrt{P_{S_{m}}}h_{S_{m}E}s+n_{E},$$

where $h_{S_{m}E}∼\mathrm{CN}\left(0,\lambda_{S_{m}E}\right)$ and $n_{E}∼\mathrm{CN}\left(0,\sigma_{E}^{2}\right)$.

Hence, from Equations (1) and (2), the received signal-to-noise ratios (SNRs) at $R_{n}$ and E in the broadcasting phase can be expressed as:

(3) $$\gamma_{S_{m}R_{n}}=\frac{P_{S_{m}}{\left|h_{S_{m}R_{n}}\right|}^{2}}{\sigma_{R_{n}}^{2}},\;\gamma_{S_{m}E}=\frac{P_{S_{m}}{\left|h_{S_{m}E}\right|}^{2}}{\sigma_{E}^{2}}.$$

#### 2.1.2. Forwarding Phase

In the forwarding phase, the decode-and-forward (DF) protocol [27] is adopted at the selected relay. For the sake of simplicity, we assume that the relay always successfully decodes the source signal. Similar to the broadcasting phase, the relaying signal from $R_{n}$ to D is also intercepted by E. Thus, the received signal at D and the overheard signal at E in the forwarding phase can be written as:

(4) $$\begin{array}{cc} y_{R_{n}D} & =\sqrt{P_{R_{n}}}h_{R_{n}D}\hat{s}+n_{D}, \end{array}$$

(5) $$\begin{array}{cc} y_{R_{n}E} & =\sqrt{P_{R_{n}}}h_{R_{n}E}\hat{s}+n_{E}, \end{array}$$

respectively, where $P_{R_{n}}$ denotes the transmit power of $R_{n}$, $\hat{s}$ denotes the re-encoded version of the source signal $\left(E[|\hat{s}{|}^{2}]=1\right)$, $h_{R_{n}D}∼\mathrm{CN}\left(0,\lambda_{R_{n}D}\right)$, $h_{R_{n}E}∼\mathrm{CN}\left(0,\lambda_{R_{n}E}\right)$ and $n_{D}∼\mathrm{CN}\left(0,\sigma_{D}^{2}\right)$.

The received SNRs at $R_{n}$ and E in the forwarding phase can be expressed as:

(6) $$\gamma_{R_{n}D}=\frac{P_{R_{n}}{\left|h_{R_{n}D}\right|}^{2}}{\sigma_{D}^{2}},\;\gamma_{R_{n}E}=\frac{P_{R_{n}}{\left|h_{R_{n}E}\right|}^{2}}{\sigma_{E}^{2}}.$$

In physical layer security, the channels between legitimate nodes, i.e., the $S_{m}\to R_{n}$ and $R_{n}\to D$ channels, are called the main channels. While the channels between legitimate nodes and the eavesdropper, i.e., the $S_{m}\to E$ and $R_{n}\to D$ channels, are called the eavesdropper channels.

Considering DF relaying transmission, the failure of the $S_{m}\to R_{n}$ or $R_{n}\to D$ transmissions will lead to the failure of the end-to-end transmission. Thus, from Equation (6), the end-to-end SNR of the main channel can be expressed as $\gamma_{S_{m}R_{n}D}^{\mathrm{main}}=\min\left\{\gamma_{S_{m}R_{n}},\gamma_{R_{n}D}\right\}$ [27,28]. Consequently, the end-to-end achievable capacity of the main channel can be expressed as:

(7) $$C_{S_{m}R_{n}D}^{\mathrm{main}}=\frac{1}{2}\log_{2}\left(1+\min\{\gamma_{S_{m}R_{n}},\gamma_{R_{n}D}\}\right),$$

where the factor 1/2 appears because the end-to-end transmission from $S_{m}$ to D is conducted in two sub-slots.

In what follows, the eavesdropper intercepts both the broadcasting and relaying signals. In this paper, we consider two well-known signal combining techniques, namely the maximal ratio combining (MRC) and selection combining (SC), at the eavesdropper.

The eavesdropper is assumed to perform the MRC technique if the selected source and the selected relay use the same codewords, i.e., repetition coding [29]. Using the MRC technique, the end-to-end received SNR of the eavesdropper channel can be written as $\gamma_{S_{m}R_{n}D}^{\mathrm{eve},\mathrm{MRC}}=\gamma_{S_{m}E}+\gamma_{R_{n}E}$. Consequently, the end-to-end achievable capacity of the eavesdropper channel can be expressed as:

(8) $$C_{S_{m}R_{n}D}^{\mathrm{eve},\mathrm{MRC}}=\frac{1}{2}\log_{2}\left(1+\gamma_{S_{m}E}+\gamma_{R_{n}E}\right).$$

The eavesdropper is assumed to employ the SC technique if the signals from the selected source and the relay are independent, i.e., the selected and relay use different codewords [30]. Using the SC technique, the end-to-end received SNR of the eavesdropper channel can be written as $\gamma_{S_{m}R_{n}D}^{\mathrm{eve},\mathrm{SC}}=\;\max\left\{\gamma_{S_{m}E},\gamma_{R_{n}E}\right\}$. Consequently, the end-to-end achievable capacity of the eavesdropper channel can be expressed as:

(9) $$C_{S_{m}R_{n}D}^{\mathrm{eve},\mathrm{SC}}=\frac{1}{2}\log_{2}\left(1+\max\left\{\gamma_{S_{m}E},\gamma_{R_{n}E}\right\}\right).$$

The system secrecy capacity can be defined by the difference between the capacity of the main channel and that of the eavesdropper channel [5,16], which can be mathematically expressed as:

(10) $$\begin{array}{cc} C_{\mathrm{secrecy}}^{\mathrm{MRC}} & =\frac{1}{2}\log_{2}\left(\frac{1+\min\{\gamma_{S_{m}R_{n}},\gamma_{R_{n}D}\}}{1+\gamma_{S_{m}E}+\gamma_{R_{n}E}}\right), \end{array}$$

(11) $$\begin{array}{cc} C_{\mathrm{secrecy}}^{\mathrm{SC}} & =\frac{1}{2}\log_{2}\left(\frac{1+\min\{\gamma_{S_{m}R_{n}},\gamma_{R_{n}D}\}}{1+\max\{\gamma_{S_{m}E},\gamma_{R_{n}E}\}}\right), \end{array}$$

for the case of using MRC and SC, respectively.

### 2.2. Source Relay Selection Process

The source relay selection process is conducted through the CSI estimation/acquisition system. Furthermore, it is noteworthy to recall that this process is carried out before the data transmission. In this paper, we propose a performance comparable, low-complexity source relay selection scheme, called the proposed source relay selection (PSRS) scheme. The PSRS scheme first selects the least vulnerable source, which minimizes the channel gains of eavesdropper channels from the sources to the eavesdropper. Thus, the best source can be selected as:

(12) $$S_{m^{*}}=\arg\min_{1\leq m\leq M}\gamma_{S_{m}E}.$$

After selecting the best source, the PSRS scheme selects the most robust relay, which maximizes the end-to-end system secrecy capacity. Hence, the best relay will be selected as:

(13) $$\begin{array}{cc} R_{n^{*}} & =\arg\max_{1\leq n\leq N}\left\{\frac{1}{2}\log_{2}\left(\frac{1+\min\{\gamma_{S_{m^{*}}R_{n}},\gamma_{R_{n}D}\}}{1+\gamma_{S_{m^{*}}E}+\gamma_{R_{n}E}}\right)\right\}, \end{array}$$

(14) $$\begin{array}{cc} R_{n^{*}} & =\arg\max_{1\leq n\leq N}\left\{\frac{1}{2}\log_{2}\left(\frac{1+\min\{\gamma_{S_{m^{*}}R_{n}},\gamma_{R_{n}D}\}}{1+\max\{\gamma_{S_{m^{*}}E},\gamma_{R_{n}E}\}}\right)\right\}, \end{array}$$

for the case of using MRC and SC, respectively.

## 3. Performance Analysis

In this section, we investigate the performance of the SRSS schemes in terms of the secrecy outage probability (SOP). Because the wireless channels undergo i.i.d. fading, for the sake of notational convenience, let $\lambda_{S_{m}R_{n}}=\lambda_{\mathrm{SR}}$, $\lambda_{S_{m}E}=\lambda_{\mathrm{SE}}$, $\lambda_{R_{n}D}=\lambda_{\mathrm{RD}}$ and $\lambda_{R_{n}E}=\lambda_{\mathrm{RE}}$. In addition, we assume that the source and the relay use the same transmit power, i.e., $P_{S_{m}}=P_{R_{n}}=P$, and all nodes have the same noise variance, i.e., $\sigma_{R_{n}}^{2}=\sigma_{D}^{2}=\sigma_{E}^{2}=\sigma^{2}$, as in [16,17,25]. It is noteworthy that the assumptions of using the same transmit powers and identical noise variances do not lose the generality of the developed analysis. Let $\bar{\gamma }=\frac{P}{\sigma^{2}}$ denote the transmit signal-to-noise ratio (SNR).

Recall that the SOP can be defined as the probability that the end-to-end achievable system secrecy capacity drops below a predefined target secrecy rate $R_{\mathrm{th}}$.

In this paper, we study the performance of the physical layer security of multi-user multirelay cooperative networks under two scenarios, namely the eavesdropper performs either the maximal-ratio combining (MRC) technique or the selection combining (SC) technique to combine overheard signals from legitimate users, i.e., the source and relay. More specifically, consider a cooperative network with multiple sources, e.g., a multiuser long-term evolution-advanced (LTE-A) cellular system [31,32]; the best user selection has been demonstrated as an efficient user scheduling to improve system performances [31,32]. On the other hand, when multiple relays are available, selecting the best relay helping the source-destination transmission can improve system throughput, e.g., for the IEEE 802.12j vehicular networks [9,33].

Studying the security issue of such networks in terms of PLS, we assume that the eavesdropper can employ either MRC or SC techniques. Please note that PLS is on the cutting-edge of technologies of security, particularly in wireless communications to prevent eavesdropping attacks [3,6]. Suppose that the source and relay use the same codeword to encode the transmitted signal, e.g., repetition coding [29]; the MRC technique can be employed by the eavesdropper, as in [34]. Otherwise, the eavesdropper may employ the SC technique, as in [35,36].

### 3.1. The Case of the Eavesdropper Using the MRC Technique

From Equation (10), the system SOP in the case when the eavesdropper performs the MRC technique can be expressed as:

(15) $$\begin{array}{cc} P_{\mathrm{SOP}}^{\mathrm{MRC}} & =\mathrm{Pr}\left(\frac{1}{2}\log_{2}\left(\frac{1+\min\{\gamma_{S_{m^{*}}R_{n^{*}}},\gamma_{R_{n^{*}}D}\}}{1+\gamma_{S_{m^{*}}E}+\gamma_{R_{n^{*}}E}}\right)<R_{\mathrm{th}}\right)\\ & =\mathrm{Pr}\left(\frac{1+\min\{\gamma_{S_{m^{*}}R_{n^{*}}},\gamma_{R_{n^{*}}D}\}}{1+\gamma_{S_{m^{*}}E}+\gamma_{R_{n^{*}}E}}<\gamma_{\mathrm{th}}\right), \end{array}$$

where $\gamma_{\mathrm{th}}=2^{2R_{\mathrm{th}}}$ represents the secrecy SNR threshold. From Equation (13) and since all of the wireless channels are assumed to be independent, Equation (15) can be rewritten as:

(16) $$P_{\mathrm{SOP}}^{\mathrm{MRC}}=\mathrm{Pr}\left(\max_{1\leq n\leq N}\left\{\frac{1+\bar{\gamma }\min\{|h_{S_{m^{*}}R_{n}}{|}^{2},|h_{R_{n}D}{|}^{2}\}}{1+\bar{\gamma }|h_{S_{m^{*}}E}{|}^{2}+\bar{\gamma }{\left|h_{R_{n}E}\right|}^{2}}\right\}<\gamma_{\mathrm{th}}\right).$$

As we can observe that the events of the probability in Equation (16) are not mutually exclusive because they include the same component $|h_{S_{m^{*}}E}{|}^{2}$, therefore conditioning on $|h_{S_{m^{*}}E}{|}^{2}=z$, the $P_{\mathrm{SOP}}^{\mathrm{MRC}}$ can be re-expressed as:

(17) $$P_{\mathrm{SOP}}^{\mathrm{MRC}}=\int_{0}^{\infty }\prod_{n=1}^{N}\underset{\Xi }{\underset{︸}{\mathrm{Pr}\left(\min\{|h_{S_{m^{*}}R_{n}}{|}^{2},|h_{R_{n}D}{|}^{2}\}<\frac{\left(\gamma_{\mathrm{th}}-1\right)}{\bar{\gamma }}+\gamma_{\mathrm{th}}z+\gamma_{\mathrm{th}}{\left|h_{R_{n}E}\right|}^{2}\right)}}f_{|h_{S_{m^{*}}E}{|}^{2}}\left(z\right)dz.$$

Since the PSRS scheme first selects the best source, the statistical characteristic of the $|h_{S_{m^{*}}E}{|}^{2}$ will be presented in the following Lemma.

**Lemma** **1.** *Suppose that* $|h_{S_{m^{*}}E}{|}^{2}=\min_{1\leq m\leq M}{\left|h_{S_{m}E}\right|}^{2}$*; the cumulative distribution function (CDF) and probability density function (PDF) of* $|h_{S_{m^{*}}E}{|}^{2}$ *can be expressed as:*

(18) $$\begin{array}{c} F_{|h_{S_{m^{*}}E}{|}^{2}}\left(z\right)=1-e^{-\frac{Mz}{\lambda_{\mathrm{SE}}}} \end{array}$$

(19) $$\begin{array}{c} f_{|h_{S_{m^{*}}E}{|}^{2}}\left(z\right)=\frac{M}{\lambda_{\mathrm{SE}}}e^{-\frac{Mz}{\lambda_{\mathrm{SE}}}} \end{array}$$

*respectively.*

**Proof.**  From Equation (12), the CDF of $|h_{S_{m^{*}}E}{|}^{2}$ can be written as:

(20) $$\begin{array}{cc} F_{|h_{S_{m^{*}}E}{|}^{2}}\left(z\right) & =\mathrm{Pr}\left(\min_{1\leq m\leq M}{\left|h_{S_{m}E}\right|}^{2}<z\right)\\ & =1-\mathrm{Pr}\left(\min_{1\leq m\leq M}{\left|h_{S_{m}E}\right|}^{2}\geq z\right) \end{array}$$

Since the sources are assumed to be independent, $F_{|h_{S_{m^{*}}E}{|}^{2}}\left(z\right)$ in Equation (20) can be further expressed as:

(21) $$\begin{array}{cc} F_{|h_{S_{m^{*}}E}{|}^{2}}\left(z\right) & =1-\prod_{m=1}^{M}\left(1-\mathrm{Pr}\left(|h_{S_{m}E}{|}^{2}<z\right)\right)\\ & =1-e^{-\frac{Mz}{\lambda_{\mathrm{SE}}}}. \end{array}$$

By taking the derivative of the right-hand side of Equation (21), the PDF of $|h_{S_{m^{*}}E}{|}^{2}$ can be obtained as in Equation (19). This completes the proof of Lemma 1. ☐

The statistical characteristic of the gain of the channel from the selected source to an arbitrary relay, i.e., $|h_{S_{m^{*}}R_{n}}{|}^{2}$, will be presented in the next Lemma.

**Lemma** **2.** *Given the selected source* $S_{m^{*}}$, the CDF and PDF of $|h_{S_{m^{*}}R_{n}}{|}^{2}$ *can be expressed as:*

(22) $$\begin{array}{c} F_{|h_{S_{m^{*}}R_{n}}{|}^{2}}\left(x\right)=1-e^{-\frac{x}{\lambda_{\mathrm{SR}}}}, \end{array}$$

(23) $$\begin{array}{c} f_{|h_{S_{m^{*}}R_{n}}{|}^{2}}\left(x\right)=\frac{1}{\lambda_{\mathrm{SR}}}e^{-\frac{x}{\lambda_{\mathrm{SR}}}}, \end{array}$$

*respectively.*

**Proof.**  Using the total probability theory [37], the CDF of $|h_{S_{m^{*}}R_{n}}{|}^{2}$ can be expressed as:

(24) $$F_{|h_{S_{m^{*}}R_{n}}{|}^{2}}\left(x\right)=\sum_{m=1}^{M}\underset{\Omega }{\underset{︸}{\mathrm{Pr}(S_{m^{*}}=S_{m})}}\mathrm{Pr}(|h_{S_{m}R_{n}}{|}^{2}<x).$$

From Equation (12), Ω in Equation (24) can be expressed as:

(25) $$\begin{array}{cc} \Omega & =\mathrm{Pr}\left(\overset{M}{\underset{\begin{array}{c} l=1\\ l\neq m \end{array}}{⋂}}\left(\gamma_{S_{l}R_{n}}>\gamma_{S_{m}R_{n}}\right)\right)\\ & =\mathrm{Pr}\left(\overset{M}{\underset{\begin{array}{c} l=1\\ l\neq m \end{array}}{⋂}}\left(|h_{S_{l}R_{n}}{|}^{2}>{\left|h_{S_{m}R_{n}}\right|}^{2}\right)\right). \end{array}$$

By conditioning on $|h_{S_{m}R_{n}}{|}^{2}=v$ and since the sources are assumed to be independent, Equation (25) can be further expressed as:

(26) $$\begin{array}{cc} \Omega & =\int_{0}^{\infty }\mathrm{Pr}(\overset{M}{\underset{\begin{array}{c} l=1\\ l\neq m \end{array}}{⋂}}\left(|h_{S_{l}R_{n}}{|}^{2}>v\right)f_{|h_{S_{m}R_{n}}{|}^{2}}\left(v\right)dv\\ & =\int_{0}^{\infty }\prod_{\begin{array}{c} l=1\\ l\neq m \end{array}}^{M}\left[1-\mathrm{Pr}(|h_{S_{l}R_{n}}{|}^{2}<v)\right]f_{|h_{S_{m}R_{n}}{|}^{2}}\left(v\right)dv \end{array}$$

and after some algebraic manipulations, Ω can be obtained as:

(27) $$\Omega =\int_{0}^{\infty }e^{\frac{(M-1)v}{\lambda_{\mathrm{SR}}}}\frac{e^{\frac{v}{\lambda_{\mathrm{SR}}}}}{\lambda_{\mathrm{SR}}}dv=\frac{1}{M}.$$

By plugging Equation (27) into (24) and after some calculation steps, the CDF and PDF of $|h_{S_{m^{*}}R_{n}}{|}^{2}$ can be obtained as presented in Equations (22) and (23), respectively. This completes the proof of Lemma 2. ☐

For the sake of notational convenience, let $X≜|h_{S_{m^{*}}R_{n}}{|}^{2}$, $Y≜|h_{R_{n}D}{|}^{2}$, $Z≜|h_{S_{m^{*}}E}{|}^{2}$ and $T≜|h_{R_{n}E}{|}^{2}$. The probability Ξ in Equation (16) can be rewritten as:

(28) $$\Xi =\mathrm{Pr}\left(\min\left\{X,Y\right\}<\frac{\gamma_{\mathrm{th}}-1}{\bar{\gamma }}+\gamma_{\mathrm{th}}z+\gamma_{\mathrm{th}}T\right).$$

The following lemma will help facilitate the derivation of Ξ.

**Lemma** **3.** *Let* U≜min{X,Y}*; the CDF of U can be expressed as:*

(29) $$F_{U}\left(u\right)=1-e^{-\frac{\lambda_{\mathrm{SR}}+\lambda_{\mathrm{RD}}}{\lambda_{\mathrm{SR}}\lambda_{\mathrm{RD}}}u}.$$

**Proof.**  The CDF of *U* can be written as:

(30) $$\begin{array}{cc} F_{U}\left(u\right) & =\mathrm{Pr}(\min\{X,Y\}<u)\\ & =1-\left[1-\mathrm{Pr}(X<u)\right]\left[1-\mathrm{Pr}(Y<u)\right]\\ & =1-e^{-\frac{u}{\lambda_{\mathrm{SR}}}}e^{-\frac{u}{\lambda_{\mathrm{RD}}}}. \end{array}$$

This completes the proof of Lemma 3. ☐

Let $\mu ≜\frac{\gamma_{\mathrm{th}}-1}{\bar{\gamma }}$ and $\theta ≜\frac{\lambda_{\mathrm{SR}}+\lambda_{\mathrm{RD}}}{\lambda_{\mathrm{SR}}\lambda_{\mathrm{RD}}}$. By conditioning on T=t and applying Lemma 3, Ξ in Equation (28) can be obtained as:

(31) $$\begin{array}{cc} \Xi & =\int_{0}^{\infty }\left[1-e^{-\theta (\mu +\gamma_{\mathrm{th}}z+\gamma_{\mathrm{th}}t)}\right]\frac{1}{\lambda_{\mathrm{RE}}}e^{-\frac{t}{\lambda_{\mathrm{RE}}}}dt\\ & =1-\frac{1}{\lambda_{\mathrm{RE}}}\int_{0}^{\infty }e^{-\mu \theta -\theta \gamma_{\mathrm{th}}z-\frac{\theta \gamma_{\mathrm{th}}\lambda_{\mathrm{RE}}+1}{\lambda_{\mathrm{RE}}}t}dt\\ & =1-\frac{e^{-\mu \theta -\theta \gamma_{\mathrm{th}}z}}{\theta \gamma_{\mathrm{th}}\lambda_{\mathrm{RE}}+1}. \end{array}$$

By plugging Equation (31) into (16) and making use of the fact that 1−e−a−bxcN=∑k=0NNk(−1)kcke−ka−kbx ([38] Equation (1.111)), the $P_{\mathrm{SOP}}^{\mathrm{MRC}}$ can be further expressed as:

(32) PSOPMRC=∫0∞∑k=0NNk(−1)k(θγthλRE+1)ke−kμθ−kθγthzMλSEeMzλSEdz,

and after some algebraic manipulations, the system SOP in the case of using MRC can be obtained as:

(33) PSOPMRC=M∑k=0NNk(−1)ke−kμθ(θγthλRE+1)k(kθγthλSE+M).

### 3.2. The Case of the Eavesdropper Using SC Technique

From Equation (11), the system SOP in the case when the eavesdropper performs the SC technique can be expressed as:

(34) $$\begin{array}{cc} P_{\mathrm{SOP}}^{\mathrm{SC}} & =\mathrm{Pr}\left(\frac{1}{2}\log_{2}\left(\frac{1+\min\{\gamma_{S_{m^{*}}R_{n^{*}}},\gamma_{R_{n^{*}}D}\}}{1+\max\{\gamma_{S_{m^{*}}E},\gamma_{R_{n^{*}}E}\}}\right)<R_{\mathrm{th}}\right)\\ & =\mathrm{Pr}\left(\frac{1+\min\{\gamma_{S_{m^{*}}R_{n^{*}}},\gamma_{R_{n^{*}}D}\}}{1+\max\{\gamma_{S_{m^{*}}E},\gamma_{R_{n^{*}}E}\}}<\gamma_{\mathrm{th}}\right), \end{array}$$

From Equation (14), Equation (34) can be rewritten as:

(35) $$P_{\mathrm{SOP}}^{\mathrm{SC}}=\mathrm{Pr}\left(\max_{1\leq n\leq N}\left\{\frac{1+\bar{\gamma }\min\{|h_{S_{m^{*}}R_{n}}{|}^{2},|h_{R_{n}D}{|}^{2}\}}{1+\bar{\gamma }\min\{|h_{S_{m^{*}}E}{|}^{2},|h_{R_{n}E}{|}^{2}\}}\right\}<\gamma_{\mathrm{th}}\right).$$

Similarly to Equation (15), the events of the probability in Equation (35) are not mutually exclusive because they include the same component $|h_{S_{m^{*}}E}{|}^{2}$. Therefore, conditioning on $|h_{S_{m^{*}}E}{|}^{2}=z$, the $P_{\mathrm{SOP}}^{\mathrm{SC}}$ can be re-expressed as:

(36) $$P_{\mathrm{SOP}}^{\mathrm{SC}}=\int_{0}^{\infty }\prod_{n=1}^{N}\underset{\Psi }{\underset{︸}{\mathrm{Pr}\left(\min\{|h_{S_{m^{*}}R_{n}}{|}^{2},|h_{R_{n}D}{|}^{2}\}<\frac{\left(\gamma_{\mathrm{th}}-1\right)}{\bar{\gamma }}+\gamma_{\mathrm{th}}\max\{z,|h_{R_{n}E}{|}^{2}\}\right)}}f_{|h_{S_{m^{*}}E}{|}^{2}}\left(z\right)dz,$$

By conditioning on T=t, Ψ can be expressed as:

(37) $$\begin{array}{cc} \Psi & =\int_{0}^{\infty }\mathrm{Pr}\left(\min\left\{X,Y\right\}<\frac{\gamma_{\mathrm{th}}-1}{\bar{\gamma }}+\gamma_{\mathrm{th}}\max\left\{z,T\right\}\right)f_{T}\left(t\right)dt\\ & =\int_{0}^{\infty }\left[\mathrm{Pr}\left(\min\left\{X,Y\right\}<\frac{\gamma_{\mathrm{th}}-1}{\bar{\gamma }}+\gamma_{\mathrm{th}}z|z>t\right)+\mathrm{Pr}\left(\min\left\{X,Y\right\}<\frac{\gamma_{\mathrm{th}}-1}{\bar{\gamma }}+\gamma_{\mathrm{th}}t|t>z\right)\right]f_{T}\left(t\right)dt\\ & =\underset{\Psi_{1}}{\underset{︸}{\int_{0}^{z}\mathrm{Pr}\left(\min\left\{X,Y\right\}<\frac{\gamma_{\mathrm{th}}-1}{\bar{\gamma }}+\gamma_{\mathrm{th}}z\right)f_{T}\left(t\right)dt}}+\underset{\Psi_{2}}{\underset{︸}{\int_{z}^{\infty }\mathrm{Pr}\left(\min\left\{X,Y\right\}<\frac{\gamma_{\mathrm{th}}-1}{\bar{\gamma }}+\gamma_{\mathrm{th}}t\right)f_{T}\left(t\right)dt}}, \end{array}$$

where $\Psi_{1}$ can be derived as:

(38) $$\begin{array}{cc} \Psi_{1} & =\frac{1}{\lambda_{\mathrm{RE}}}\int_{0}^{z}\left[1-e^{-\mu (\theta -\gamma_{\mathrm{th}}z)}\right]e^{-\frac{1}{\lambda_{\mathrm{RE}}}t}dt\\ & =\frac{1}{\lambda_{\mathrm{RE}}}\int_{0}^{z}e^{-\frac{1}{\lambda_{\mathrm{RE}}}t}dt-\frac{1}{\lambda_{\mathrm{RE}}}\int_{0}^{z}e^{-\mu \theta -\theta \gamma_{\mathrm{th}}z-\frac{1}{\lambda_{\mathrm{RE}}}t}dt\\ & =1-e^{-\frac{1}{\lambda_{\mathrm{RE}}}z}-e^{-\mu \theta -\theta \gamma_{\mathrm{th}}z}+e^{-\mu \theta -\frac{\theta \gamma_{\mathrm{th}}\lambda_{\mathrm{RE}}+1}{\lambda_{\mathrm{RE}}}z}, \end{array}$$

and $\Psi_{2}$ can be derived as:

(39) $$\begin{array}{cc} \Psi_{2} & =\frac{1}{\lambda_{\mathrm{RE}}}\int_{z}^{\infty }e^{-\frac{1}{\lambda_{\mathrm{RE}}}t}dt-\frac{1}{\lambda_{\mathrm{RE}}}\int_{z}^{\infty }e^{-\mu \theta -\theta \gamma_{\mathrm{th}}t-\frac{1}{\lambda_{\mathrm{RE}}}t}dt\\ & =e^{-\frac{1}{\lambda_{\mathrm{RE}}}z}-\frac{1}{\theta \gamma_{\mathrm{th}}\lambda_{\mathrm{RE}}+1}e^{-\mu \theta -\frac{\theta \gamma_{\mathrm{th}}\lambda_{\mathrm{RE}}+1}{\lambda_{\mathrm{RE}}}z}. \end{array}$$

Now, plugging Equations (38) and (39) into (37) and after some manipulations, $\Xi_{SC}$ can be obtained as:

(40) $$\begin{array}{cc} \Psi & =1-e^{-\mu \theta -\theta \gamma_{\mathrm{th}}z}+\frac{\theta \gamma_{\mathrm{th}}\lambda_{\mathrm{RE}}}{\theta \gamma_{\mathrm{th}}\lambda_{\mathrm{RE}}+1}e^{-\mu \theta -\frac{\theta \gamma_{\mathrm{th}}\lambda_{\mathrm{RE}}+1}{\lambda_{\mathrm{RE}}}z}. \end{array}$$

Finally, substituting Equation (40) into Equation (36), $P_{\mathrm{SOP}}^{\mathrm{SC}}$ is obtained as:

(41) $$P_{\mathrm{SOP}}^{\mathrm{SC}}=\frac{M}{\lambda_{\mathrm{SE}}}\int_{0}^{\infty }{\left[1-e^{-\mu \theta -\theta \gamma_{\mathrm{th}}z}+\frac{\theta \gamma_{\mathrm{th}}\lambda_{\mathrm{RE}}}{\theta \gamma_{\mathrm{th}}\lambda_{\mathrm{RE}}+1}e^{-\mu \theta -\frac{\theta \gamma_{\mathrm{th}}\lambda_{\mathrm{RE}}+1}{\lambda_{\mathrm{RE}}}z}\right]}^{N}e^{-\frac{M}{\lambda_{\mathrm{SE}}}z}dz.$$

In order to further simplify the integral (41), we rely on the trinomial coefficient, i.e., ${\left(a+b+c\right)}^{n}$ = ∑i,j,kn=i+j+kni,j,kaibjck [39]. Consequently, the $P_{\mathrm{SOP}}^{\mathrm{SC}}$ can be expressed as:

(42) PSOPSC=MλSE∫0∞∑i,j,k≥0NNi,j,k(−1)je−jθ(μ+γthz)θγthλREθγthλRE+1ke−k(μθ+θγthλRE+1λREz)e−MλSEzdz=MλSE∑i,j,k≥0NNi,j,k(−1)jθγthλREθγthλRE+1ke−(j+k)μθ∫0∞e−(jθγth+kθγthλRE+1λRE+MλRE)zdz,

and after some algebraic manipulations, the $P_{\mathrm{SOP}}^{\mathrm{SC}}$ can be obtained as:

(43) PSOPSC=MλSE∑i,j,kNNi,j,k(−1)jθλREγthθλREγth+1ke−(j+k)μθjθγth+k(θγthλRE+1)/λRE+M/λSE.

## 4. Numerical Results and Discussions

In this section, representative numerical results are provided to illustrate the secrecy performance of the PSRS scheme in terms of the secrecy outage probability (SOP). Insightful discussions relating to the impacts of the main-to-eavesdropper ratio (MER), the signal-to-noise ratio (SNR), the source relay distance, $d_{\mathrm{SR}}$, the secrecy target data rate, $R_{\mathrm{th}}$, and the numbers of sources and relays on the system SOP will be presented. Additionally, performance comparison, as well as the complexity order between the proposed scheme, the OJSRS scheme and the random source relay selection (RSRS) scheme are provided to show the advantages and disadvantages of our proposed scheme.

Without loss of generality, we consider a line network that is deployed in a unit squared area; in particular, the sources are located at the same place and so are the relays, which is often used in the literature [16,17,18,21,23,25]. Please note that distances between the sources and between the relays are, respectively, considered the same, which leads to the corresponding average channel gains being the same. However, the instantaneous channel gains are still random; therefore, these are the order statistics for the minimum and maximum of the channel gains, which satisfy Equations (12)–(14). The average channel gain is modeled as $\lambda_{\mathrm{XY}}=d_{\mathrm{XY}}^{-\epsilon }$, where *d* represents the Euclidean distance and *ϵ* stands for path-loss exponent. Herein, we set ϵ=4 (for an urban environment). The main-to-eavesdropper ratio (MER) can be defined as the ratio of the average main channel gain over the average eavesdropper channel gain in one hop [16,25], i.e., ${\mathrm{MER}}_{1}=\frac{\lambda_{\mathrm{SR}}}{\lambda_{\mathrm{SE}}}$ and ${\mathrm{MER}}_{2}=\frac{\lambda_{\mathrm{RD}}}{\lambda_{\mathrm{RE}}}$. Without loss of generality, in our simulation, we set ${\mathrm{MER}}_{1}={\mathrm{MER}}_{2}=\mathrm{MER}$. Recall that, in this section, SNR stands for $\bar{\gamma }=\frac{P}{\sigma^{2}}$.

**Figure 2:**
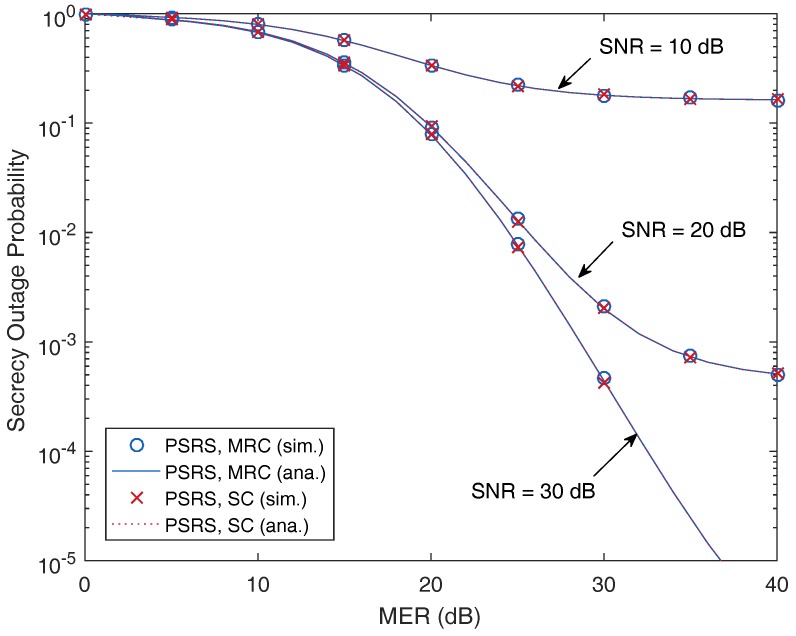
Secrecy outage probability as a function of the main-to-eavesdropper ratio (MER) with different values of the signal-to-noise ratio (SNR), where M=3, N=3, $d_{\mathrm{SR}}=0.5$ and $R_{\mathrm{th}}\;=\;3$ bits/s/Hz.

In Figure 2, we plot the SOP as a function of MER with different values of SNR. As can be seen, the SOP decreases along with the increasing of MER, which means that the transmission is more secure if the legitimate channels have better conditions than that of the eavesdropper channels. More specifically, in Figure 2, two parameters are varied, i.e., the main-to-eavesdropper ratio (MER) and the signal-to-noise ratio (SNR). For a given value of SNR, increasing MER leads to decreasing of the SOP. Furthermore, for a given value of MER, the SOP decreases as the SNR increases. The latter result can be explained as follows. The MER can be defined as the ratio of the average channel gains between the main channel and the eavesdropper channel [4]. However, in practice, the eavesdropper channel gain could not be controlled by legitimate nodes. Therefore, in order to improve the MER, one solution is to improve the channel gain of the main channel (or move far away eavesdroppers to degrade the eavesdropper channel gain). Additionally, one of the typical methods to achieve better SNR of the main channel is to increase the transmit power of the source. Hence, increasing the transmit power of the sources also helps improve the system secrecy.

**Figure 3:**
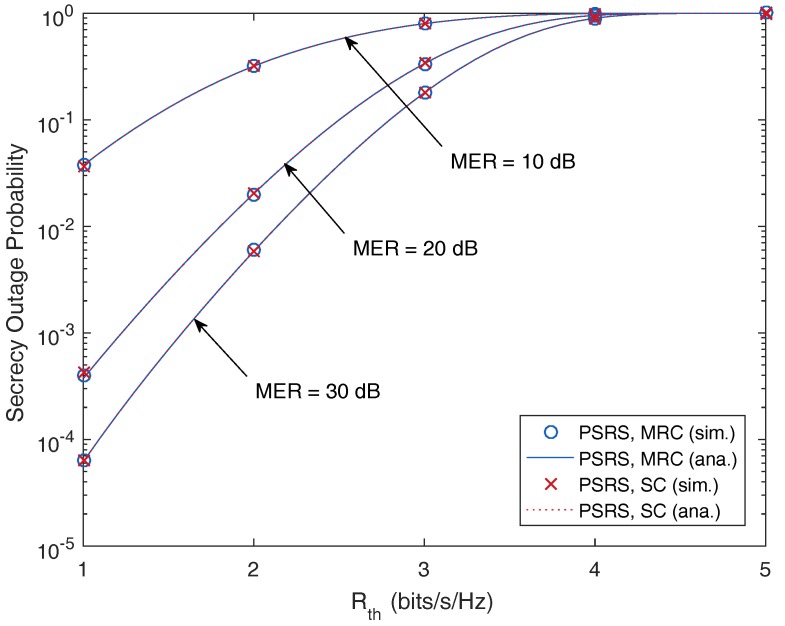
Secrecy outage probability as a function of the secrecy target data rate, $R_{\mathrm{th}}$ (bits/s/Hz), with different values of the main-to-eavesdropper ratio (MER), where M=3, N=3, $d_{\mathrm{SR}}=0.5$ and SNR =10 dB.

Figure 3 presents the SOP as a function of the secrecy target data rate, $R_{\mathrm{th}}$ (bits/s/Hz), with different values of MER. As can be seen, the SOP increases as $R_{\mathrm{th}}$ increases. This means that if the sources and/or the relays are allowed to transmit with a higher secrecy data rate (in order to obtain higher throughput), the relaying transmission will be more vulnerable to the eavesdropper.

**Figure 4:**
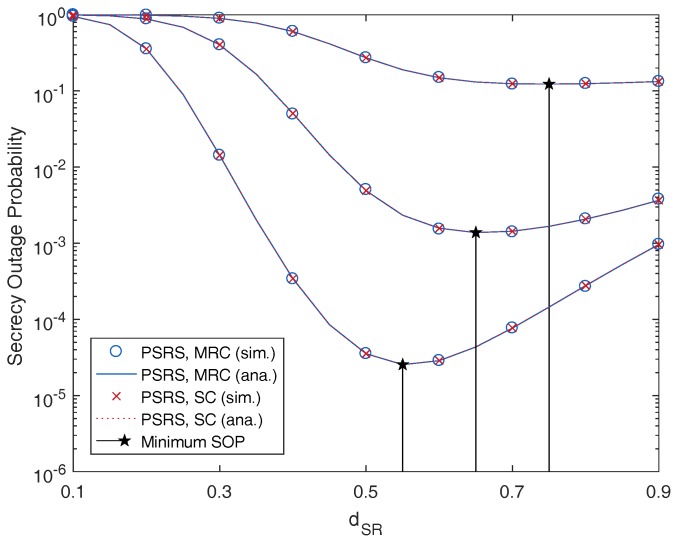
Secrecy outage probability as a function of the source relay distance, $d_{\mathrm{SR}}$, with different values of the main-to-eavesdropper ratio (MER), where M=3, N=3, SNR =20 dB and $R_{\mathrm{th}}=2$ bits/s/Hz.

In Figure 4, we plot the SOP as a function of the distance between the sources and the relays, $d_{\mathrm{SR}}$. We can observe that the SOP is a convex function with respect to $d_{\mathrm{SR}}$. Therefore, from the derived SOP, the optimal position of the selected relay that minimizes the SOP can be numerically found. For example, with our simulation setting, the SOP is minimum when $d_{\mathrm{SR}}=0.75,0.65,0.55$ for MER=10,20,30 dB, respectively.

There actually is a performance gap between the MRC and SC techniques. In particular, we present the performance gap between the two techniques in Table 1 using the analysis results with SNR = 30 dB, M=3, N=3, $d_{\mathrm{SR}}=0.5$ and $R_{\mathrm{th}}=3$ bits/s/Hz.

As shown in Table 1, the secrecy outage probability (SOP) in the case of the eavesdropper using MRC is larger than that of the case of using SC. This means that the relaying system is more vulnerable when the eavesdropper uses MRC compared to the case of using SC. The reason is that with the MRC technique, the eavesdropper is able to collect more information of the transmit data than using the SC technique. However, the eavesdropper can only employ the MRC technique when the source and relay use the same codeword to encode the transmitted signal, e.g., repetition coding [29]. Otherwise, the eavesdropper employs the SC technique [26,36], which is to choose the strongest overheard signal among that transmitted from the source or relay.

Because this performance gap is too small, it is hard to be recognized in the plots of our paper, and these differences of the SOP between the MRC and SC techniques can be ignored. Therefore, Figure 2, Figure 3 and Figure 4 show similar performances achieved by the MRC and SC techniques.

For a benchmark comparison of the PSRS scheme, we now present the selection criterion of the optimal joint source relay selection (OJSRS) scheme. Let $S_{m^{*}}$ and $R_{n^{*}}$ denote the selected source and relay, respectively, in each transmission block. The OJSRS scheme aims to select a pair of source relay nodes that maximizes the end-to-end system secrecy capacity. Mathematically, the selection criterion of the OJSRS scheme can be expressed as:

(44) $$\begin{array}{cc} \left(S_{m^{*}},R_{n^{*}}\right) & =\arg\max_{1\leq m\leq M}\max_{1\leq n\leq N}\left\{\frac{1}{2}\log_{2}\left(\frac{1+\min\{\gamma_{S_{m}R_{n}},\gamma_{R_{n}D}\}}{1+\gamma_{S_{m}E}+\gamma_{R_{n}E}}\right)\right\}, \end{array}$$

(45) $$\begin{array}{cc} \left(S_{m^{*}},R_{n^{*}}\right) & =\arg\max_{1\leq m\leq M}\max_{1\leq n\leq N}\left\{\frac{1}{2}\log_{2}\left(\frac{1+\min\{\gamma_{S_{m}R_{n}},\gamma_{R_{n}D}\}}{1+\max\{\gamma_{S_{m}E},\gamma_{R_{n}E}\}}\right)\right\}, \end{array}$$

for the case of using MRC and SC, respectively. To the best of the authors’ knowledge, such a selection criterion (which takes into account the main channel, as well as the eavesdropper channel) has not been investigated in the literature since its performance analysis is intractable.

**Figure 5:**
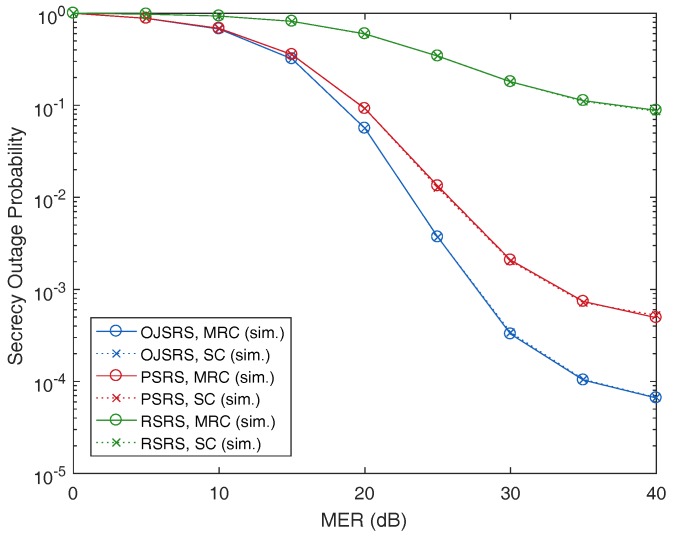
Performance comparison between the proposed source relay selection (PSRS), the optimal joint source relay selection (OJSRS) and the random source relay selection (RSRS) schemes with the secrecy outage probability as a function of the main-to-eavesdropper ratio (MER) with M=3, N=3, $d_{\mathrm{SR}}=0.5$, SNR =20 dB and $R_{\mathrm{th}}=3$ bits/s/Hz.

**Figure 6:**
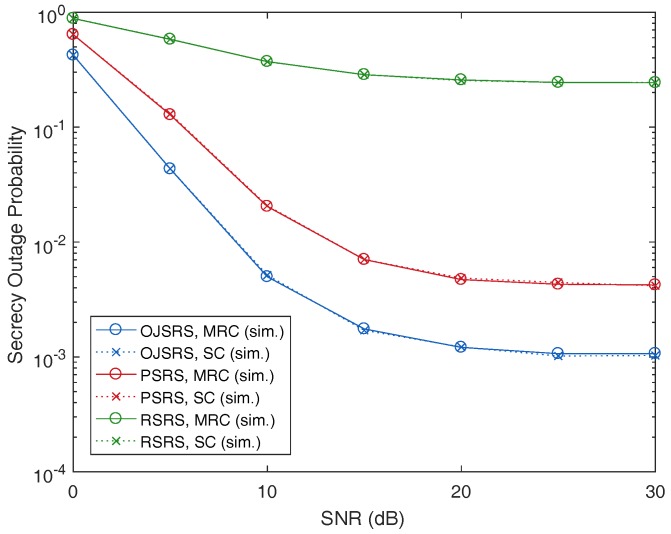
Performance comparison between the PSRS, OJSRS and RSRS schemes with the secrecy outage probability as a function of signal-to-noise ratio (SNR) with M=3, N=3, $d_{\mathrm{SR}}=0.5$, MER =20 dB and $R_{\mathrm{th}}=2$ bits/s/Hz.

Performance comparisons between the PSRS scheme, the OJSRS scheme and the random source relay selection (RSRS) scheme are presented in Figure 5 and Figure 6, where we plot the SOP as a function of MER and SNR, respectively. As can be seen, on the one hand, both the PSRS and OJSRS schemes significantly outperform the RSRS scheme, which demonstrates the benefit of user selection in cooperative relaying networks. On the other hand, the PSRS scheme does not provide a better performance than that of the OJSRS scheme.

From Figure 2, Figure 3, Figure 4, Figure 5 and Figure 6, it is shown that the SOPs are the same for the cases of the MRC technique and the SC technique employed by the eavesdropper. Hence, we can conclude that both the MRC and SC techniques have the same effect on the security of the considered cooperative transmission.

**Figure 7:**
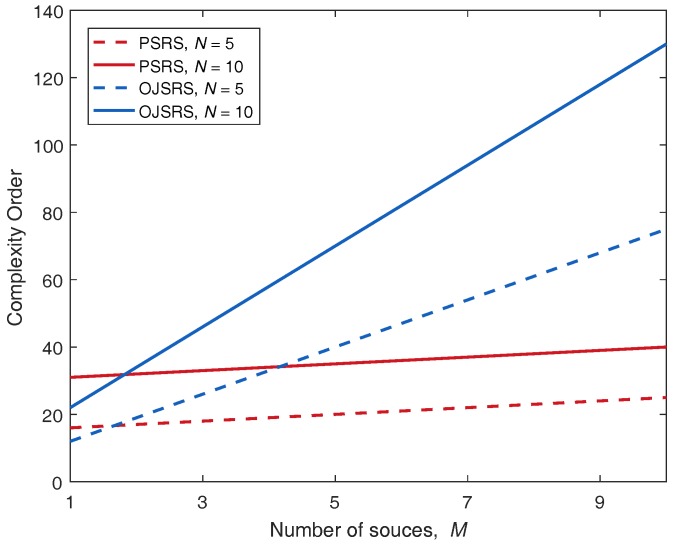
Comparison of the complexity order between the PSRS and OJSRS schemes.

In order to highlight the advantage of the PSRS scheme, we compare its operation complexity with that of the OJRDS scheme by introducing the complexity order metric. The complexity order of a selection scheme can be defined as an amount of CSI estimations (or channel estimations) that a selection criterion of a scheme (the PSRS scheme or the OJSRS scheme) needs to know to select the best source relay pair per transmission block. From Equations (12)–(14), the amount of CSI required by the PSRS scheme is M+3N. From Equations (44) and (45), the amount of CSI required by the OJSRS scheme is MN+M+2N. In Figure 7, the complexity orders of the PSRS and OJSRS schemes are evaluated. As can be seen, the complexity order of the PSRS scheme is much lower than that of the OJSRS scheme, especially when the number of relays is relatively large. This is the noteworthy advantage of the PSRS scheme. Note that the RSRS scheme does not require CSI to select the source relay pair.

**Figure 8:**
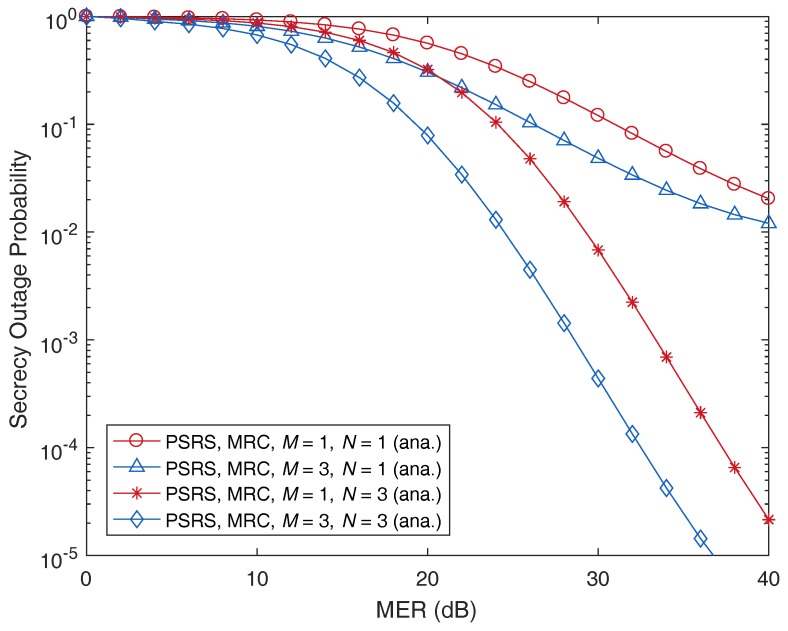
Illustration of the impact of the number of sources, *M*, and the number of relays, *N*, on the secrecy outage probability as a function of the main-to-eavesdropper ratio (MER), where $d_{\mathrm{SR}}=0.5$, SNR =30 dB and $R_{\mathrm{th}}=3$ bits/s/Hz.

**Figure 9:**
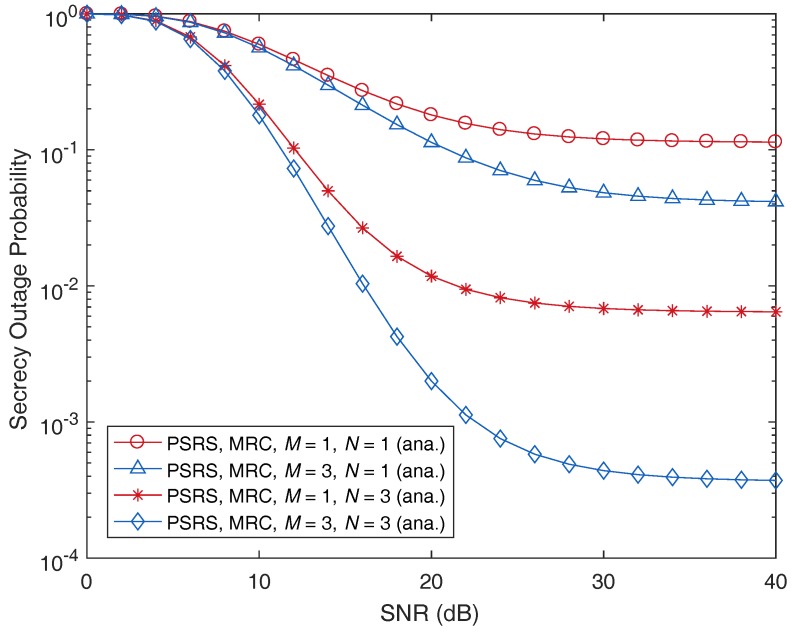
Illustration of the impact of the number of sources, *M*, and the number of relays, *N*, on the secrecy outage probability as a function of the signal-to-noise ratio (SNR), where $d_{\mathrm{SR}}=0.5$, MER =30 dB and $R_{\mathrm{th}}=3$ bits/s/Hz.

We now turn our attention to the impact of the numbers of sources and relays on the secrecy performance of the PSRS scheme. In Figure 8 and Figure 9, we plot the SOP as a function of MER and SNR, respectively, with different numbers of sources, *M*, and relays, *N*. As can be observed, when the number of sources or relays increases, the SOP decreases. This means that when more nodes participate in a cooperative relaying transmission, the security of the transmission will be improved. Please note that one possible reason for the benefit of user/relay selection is that in wireless communications, independent users have a low probability of experiencing the deep effects of fading simultaneously [40]. Therefore, if more sources and relays participate in a cooperative transmission, we have more chances to choose ones that exhibit low-correlated, better channel conditions, which leads to a better cooperative transmission and a robustness to attacks from eavesdroppers (according to information-theoretic perspectives). Thus, increasing the number of sources and relays can improve the PLS, i.e., decrease the secrecy outage probability (SOP), of cooperative relaying transmission.

Moreover, the SOP decreases more quickly when *M* is fixed and *N* is increased than *M* is increased and *N* is fixed. This observation means that the number of relays has a stronger impact on the secrecy performance compared to that of the number of sources. It can be explained as follows. Please note that we assume that the direct links between the sources and the destination are not available due to severe fading or deep shadowing as in [16,17,23]. Hence, the relays play a key role in such cooperative relaying transmissions. On the other hand, in the proposed source-relay selection scheme, the selected source is separately chosen based on the channel conditions of the links from sources to the eavesdropper, while the selected relay is chosen based on the channel conditions of the links from the selected source to the eavesdropper, from the relays to the eavesdropper, from the selected source to the relays and from the relays to the destination. Because the relays are greatly involved in the cooperative transmission with PLS compared to the sources, the number of relays therefore has more impact on the secrecy outage performance, i.e., more decreasing of the SOP, than that of the number of sources.

## 5. Conclusions

In this paper, we have investigated the secrecy performance of opportunistic scheduling in multiuser multirelay cooperative networks. We have proposed the PSRS scheme, which aims to improve the physical layer security (PLS) of the considered cooperative relaying networks. In addition, two combining techniques, namely maximal ratio combing (MRC) and selection combining (SC), have been considered at the eavesdropper. The closed-form expressions of the secrecy outage probability (SOP) have been derived and verified by the computer simulation. Our results showed that the PSRS scheme provides a reasonable secrecy outage performance compared to that of the optimal joint source relay selection (OJSRS) scheme, but significantly reduces the complexity of the selection process. Indeed, in the case of the OJSRS scheme, the complexity order is MN+M+2N, while the complexity order of the PSRS scheme is reduced to M+3N. The impact of the number of sources and relays on the SOP is also explored. We have shown that increasing the number of sources and relays can decrease the SOP of cooperative relaying transmission. Additionally, the number of relays has more impact on the secrecy outage performance, i.e., more decreasing of the SOP, than that of the number of sources. Furthermore, the PSRS scheme provides a reasonable secrecy outage performance compared to that of the optimal joint source relay selection (OJSRS) scheme. 

## Figures and Tables

**Table 1:** The differences of the secrecy outage probability with the eavesdropper using the maximal ratio combining (MRC) and selection combining (SC) techniques.

MER (dB)	0	2	4	6	8	10
MRC	1	0.962594	0.900831	0.848160	0.773983	0.673843
SC	1	0.945074	0.900156	0.848150	0.773983	0.673843
Performance gap	0	0.017520	0.000675	0.000010	0	0
